# Cotterell's *Common Injuries to Limbs*

**Published:** 1885-06

**Authors:** 


*Common Injuries to Limbs.* By E. COTTERELL, M.R.C.S. Pp. 108. London: H. K. LEWIS. 1885.

WE have nothing but good to say of this little work. It gives thoroughly trustworthy accounts of the common minor injuries to limbs, with exceedingly sensible remarks on the best modes of treating these. The common and pernicious habit of over-resting joints after injury is pointed out and condemned, and clear instructions are given to prevent the stiffening of joints that is thus begotten. Many useful hints are given for the treatment of such injuries as Colles' fracture; lawn tennis leg, elbow, and arm; fracture of the tip of the olecranon, and so forth. The remarks about bone-setting are practical and to the point. We can see that the author has well thought over as well as scientifically treated these every-day injuries, and he has produced a work which is well worthy of perusal.

